# Correlates of Presence and Remission of Post-trauma Stress Disorder in Incarcerated Women: A Case-Control Study Design

**DOI:** 10.3389/fpsyt.2021.748518

**Published:** 2021-12-08

**Authors:** Shaoling Zhong, Xiaomin Zhu, Graham Mellsop, Jiansong Zhou, Xiaoping Wang

**Affiliations:** ^1^National Clinical Research Center for Mental Disorders, Department of Psychiatry, The Second Xiangya Hospital, Central South University, Changsha, China; ^2^Hunan Key Laboratory of Psychiatry and Mental Health, Changsha, China; ^3^Suzhou Guangji Hospital, The Affiliated Guangji Hospital of Soochow University, Suzhou, China; ^4^Waikato District Health Board (DHB), Hamilton, New Zealand

**Keywords:** post-traumatic stress disorder, prison, remission, trauma, female offenders, violent crimes

## Abstract

Women in prison are vulnerable to post-trauma stress disorder (PTSD). However, little is known about the presence of PTSD in imprisoned women or of the natural course of that disorder. The purpose of this study was to assess the risk factors for PTSD in incarcerated women and document correlations of remission. We conducted a retrospective case-control study in the Female Prison of Hunan Province, China. Participants were screened for PTSD and depression using the Chinese version of the MINI International Neuropsychiatric Interview (MINI) 5.0. Of the 2,322 women screened, 220 met the criterion for PTSD on admission. Remission (*N* = 142) and non-remission PTSD (*N* = 78) were then separated depending on current PTSD status. History of drug use (OR = 0.43, 95% CI: 0.28–0.66, *p* < 0.001) and violent offense (OR = 1.56, 95% CI: 1.17–2.09, *p* < 0.001) were associated with the presence of PTSD. Positive associations with remission were found for longer length of sentence (61–120 vs. 13–60 months) (OR = 4.20, 95% CI: 1.50–11.75, *p* = 0.006), violent offense (OR = 2.50, 95% CI: 1.12–5.60, *p* = 0.03), and comorbid depression (OR = 29.69, 95% CI: 3.50–251.78, *p* = 0.002); while a negative correlate was identified for past depression (OR = 0.24, 95% CI: 0.11–0.53, *p* < 0.001). Although some incarcerated women with PTSD can spontaneously remit, this study suggested certain criminological and clinical risk factors are associated with the presence of PTSD and others with remission over time. Timely screening and effective intervention should be tailored for individuals with PTSD in prisons.

## Introduction

There are over 100,000 women imprisoned in China, with an increase rate of 39% in the last decade (1, 2). Nearly 211,870 women were incarcerated in the United States, as compared to 112,797 in 2010 (3). Approximately 7% of prisoners are women but their rate of increase is 9 times greater than for men across the world (4). High incidence of mental health problems and serious mental illness in the prison population have been identified. Incarcerated women have been shown to be particularly vulnerable to post-traumatic stress disorder (PTSD) (5, 6). Research has found a high prevalence of mental illness among incarcerated residents compared with the general population, in particular for PTSD. In 2006, an epidemiological study in a women's prison in China reported the lifetime and 12-month prevalences of PTSD were 16 and 11%, respectively. Recent national surveys suggest the prevalence in the general population in China were 0.2–0.3% (7, 8). In addition to increasing risk of mental health problems among prison population, incarcerated women have different mental health needs from men. A systematic review published in 2018 reported that the prevalence of PTSD in imprisoned women is 3 times that in men (9). Furthermore, PTSD is associated with increased risks of suicide (10, 11), reoffending (12), and addiction (13).

Exposure to traumatic events is the central diagnostic criterion of PTSD in the fifth edition of the *Diagnostic and Statistical Manual of Mental Disorders* (*DSM-V*) (14). Previous studies have shown that a high percentage of incarcerated women reported having been exposed to some type of traumatic events, such as physical, emotion, and sexual abuse, during their lifespan (15). For example, early research with 464 incarcerated women in the United States by Green et al. demonstrated that stressful life events contributed to the diagnosis of PTSD (16). There is a connection between childhood abuse and subsequent mental illness for women in prisons (17, 18). Tripodi and Davis (17) studied a random sample of incarcerated women and found that a history of childhood victimization was associated with psychological or emotional problems in adulthood. Among traumatic events, incarceration itself has been identified as a very important event to precipitate PTSD, especially for those with children (15, 19). In addition, pre-existing vulnerability factors, such as primary diagnosis of depression, may also contribute to developing PTSD in the community sample (20). Another potential risk for developing PTSD is the mismatch between women' needs and the standard male model designs of correctional facilities (21).

Although some studies have examined the prevalence of traumas, including physical, emotional, and sexual abuse, among incarcerated women during the stay in prison (5, 7, 16), little is known about the factors of PTSD at the time of incarceration. In addition, constant stress and insufficient mental health services in prison (22) make it difficult for women to seek or receive treatment. In the general population, a recent meta-analysis (23) revealed 44% of individuals could remit from PTSD without any treatment or intervention and the median time is 40 months. However, the natural course of PTSD in incarcerated women remains unknown. A better understanding of the specific trajectories for incarcerated women will allow for more targeted interventions that could promote mental health and reduce negative outcomes. Therefore, we conducted this study on (a) the risk factors for PTSD at the initiate of incarceration and (b) the correlations of spontaneous remission from PTSD among imprisoned women in China.

## Methods

### Study Sample

A retrospective study of incarcerated women from December 2012 to November 2013 in Hunan Provincial Female Prison was conducted. The study sample was recruited to assess and improve the mental health status of people in prisons and findings have been published in a previous paper (24). The total population in Hunan Provincial Female Prison during the study period was 2916. For this study, inclusion criteria were: Chinese ethnicity, able to communicate and fluent in Chinese language. Excluded were those with listening disability, speech impairment, or psychotic disorders. The project was approved by the Ethical Committees of the Second Xiangya Hospital, the Hunan Female Prison, and the Bureau of Prisons in Hunan Province.

### Measures

#### Demographic, Clinical, and Criminal Characteristics

Demographics, clinical, and criminal history were recorded using a standard form during interview, including age, education level (low: ≤9 years vs. high: >9 years), employment status prior to the prison, residence, marital status, criminal history, recent violent offense, smoking, drug use, family history of crime and mental diseases, physical diseases, length of sentence, and length of stay in prison (i.e., the duration between the date of recruitment and their first day of incarceration).

#### Diagnostic Assessment

The MINI-International Neuropsychiatric Interview (M.I.N.I.) (25) was developed by Lecrubier and Sheehan. It is a structured interview tool used to make diagnosis according to the *Diagnostic and Statistical Manual of Mental Disorders-IV* (*DSM-IV*) (26) and the tenth revision of the *International Statistical Classification of Diseases and Related Health Problems* (*ICD-10*) (27). The Chinese version 5.0 of the M.I.N.I. scale has been demonstrated to show good reliability and validity (28) and has been used in a range of populations. Lifetime and current (past month) PTSD and depression were established. Dates of presence and traumatic events were recorded. By interviewing, we assigned the diagnosis of PTSD if individuals met the criteria at the time of their incarceration. We defined the remission from PTSD in those who no longer met the criteria existence for the disorder in the most recent 4 weeks.

### Procedure

**Figure 1:**
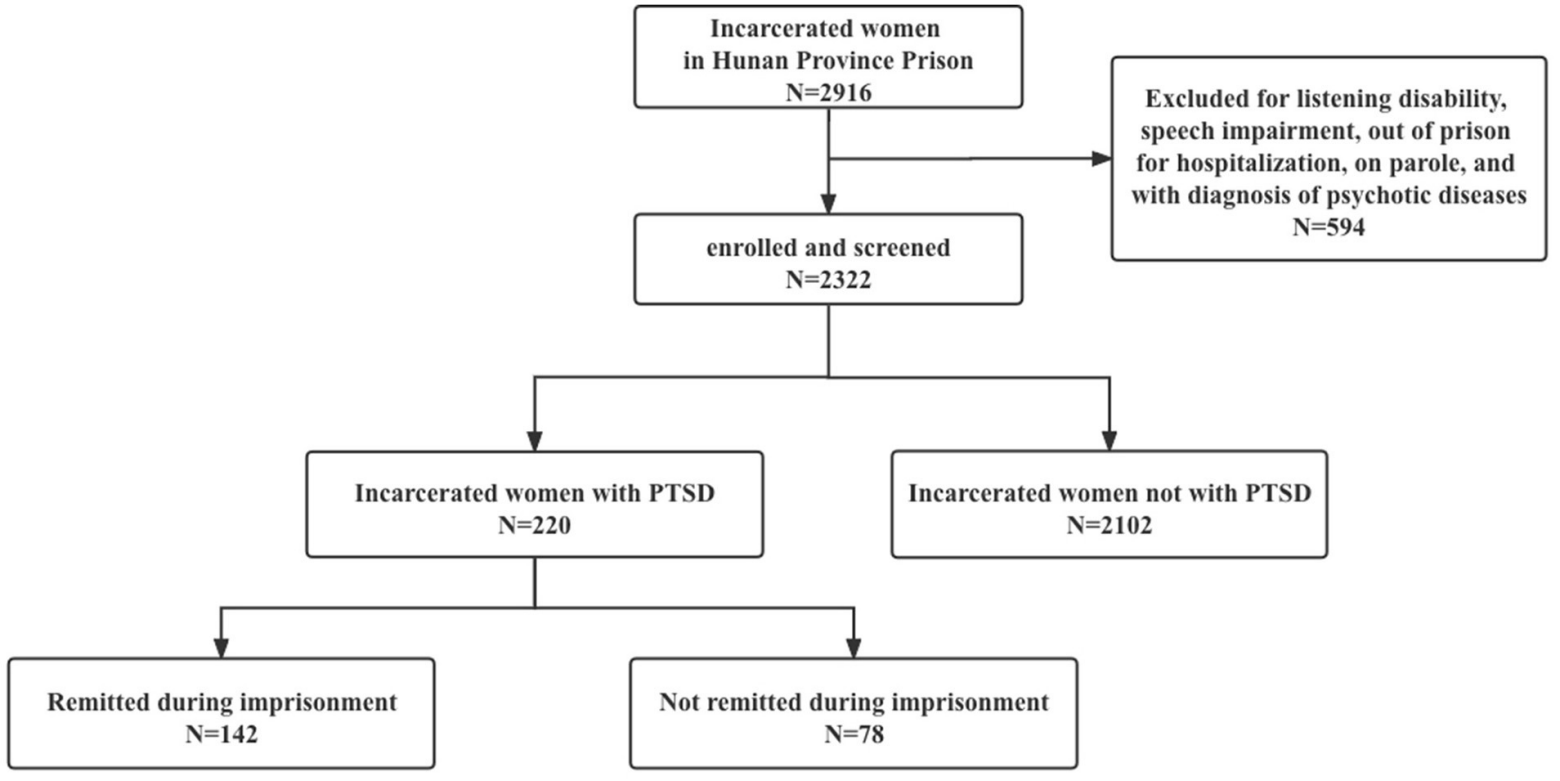
Flow-chart of the study design. PTSD, post-traumatic stress disorder.

Incarcerated women were invited to participate in the study. They were informed of the aim of the study and were further told that participation or not would not affect their routine care and management in the prison. Those who agreed to take part gave their written consent. The questionnaire of the demographics and criminal characteristics was completed in a quiet room. The criminal history was collected from the police records. The study flowchart was presented in Figure 1.

### Statistical Analysis

Mean and standard deviation (sd.) were presented for continuous variables, while numbers and percentages were presented for categories. Chi-square tests and independent-samples *t*-tests were investigated to analyze categorical and continuous variables between groups as appropriate. Then, the multivariate binary logistic regression (Likelihood Ratio method) analyses were applied to explore the independent variables between groups (healthy controls vs. individuals with PTSD). Variables that reported a *p*-value < 0.1 in the univariate analyses were entered as the independent variables in the binary analysis, while the diagnosis of PTSD was set as the dependent variable. Finally, the multivariate binary logistic (Likelihood Ratio method) regression analyses were performed to explore the remission pattern of PTSD during imprisonment. Remission from PTSD was defined as the dependent variable and variables with a *p*-value < 0.1 in univariate analyses were set as the independent variables. Age and length of stay in prison was adjusted in the model. The statistical analyses were conducted using IBM SPSS version 26.0. Two-tailed *p*-values of 0.05 were considered as statistically significant.

## Results

### Socio-Demographics, Clinical, and Criminal Characteristics of the Sample

The results of the socio-demographic, clinical, and criminal characteristics are listed in Table 1. Compared to non-PTSD, incarcerated women with PTSD were more likely to be living in rural areas (39.1 vs. 31.3%, *p* = 0.02), employed prior to the prison (65.0 vs. 56.0%, *p* = 0.01), have been violent offenders (44.1 vs. 30.0%. *p* < 0.01), non-smokers (18.2 vs. 27.7%, *p* < 0.01), no history of drug use (81.8 vs. 72.3%, *p* < 0.01), and have a past history of depression (34.5 vs. 29.0%, *p* < 0.01). Significant differences were also found in the length of sentence between PTSD and non-PTSD (*p* < 0.001).

**Table 1:** Socio-demographics, clinical, and criminological characteristics among incarcerated women.

**Variables**	**Total (*****N*** **= 2322)**		**PTSD (*****N*** **= 220)**		**Non-PTSD (*****N*** **= 2102)**		***F***	***P*-value**
	***N***	**%**	***N***	**%**	***N***	**%**		
Residence							5.62	0.02
Urban	1,579	68.0	134	60.9	1,445	68.7		
Rural	743	32.0	86	39.1	657	31.3		
Education level							0.35	0.55
Lower	1,748	75.3	162	73.6	1,586	75.5		
Upper	574	24.7	58	26.4	516	24.5		
Employment							6.58	0.01
Unemployed	1,002	43.2	77	35.0	925	77.0		
Employed	1,320	56.8	143	65.0	1,177	143.0		
Marriage							0.82	0.36
Married	1,017	43.8	90	40.9	927	44.1		
Unmarried	1,305	56.2	130	59.1	1,175	55.9		
Had kids							4.09	0.04
Absent	675	29.1	51	23.2	624	29.7		
Present	1,647	70.9	169	76.8	1,478	70.3		
Criminal history							0.74	0.39
No	2,163	93.2	208	94.5	1,955	93.0		
Yes	159	6.8	12	5.5	147	7.0		
Violent offense							18.46	<0.001
No	1,595	68.7	123	55.9	1,472	70.0		
Yes	727	31.3	97	44.1	630	30.0		
Smoking							9.18	<0.001
Absent	1,700	73.2	180	81.8	1,520	72.3		
Present	622	26.8	40	18.2	582	27.7		
Drug use history							22.85	<0.001
Absent	1,726	74.3	193	87.7	1,533	72.9		
Present	596	25.7	27	12.3	569	27.1		
Family history of crime							<0.001	0.98
Absent	1,996	86.0	189	85.9	1,807	86.0		
Present	326	14.0	31	30.9	295	14.0		
Family history of mental diseases							0.02	0.89
Absent	2,179	93.8	206	93.6	1,973	93.9		
Present	143	6.2	14	6.4	129	6.1		
Physical disease history							1.57	0.21
Absent	1,233	53.1	108	49.1	1,125	53.5		
Present	1,089	46.9	112	50.9	977	46.5		
Depression history							2.92	0.09
Absent	1,636	70.5	144	65.5	1,492	71.0		
Present	686	29.5	76	34.5	610	29.0		
Length of sentence (months)							14.76	<0.001
1–12	27	1.2	0	0.0	27	1.3		
13–60	602	25.9	47	21.4	555	26.4		
61–120	546	23.5	39	17.7	507	24.1		
>120	1,147	49.4	134	60.9	1,013	1,038.3		

*PTSD, post-traumatic stress disorder*.

### Risk Factors for the Presence of PTSD at Imprisonment

Multiple logistic regression showed that violent offense (OR: 1.56, 95% CI: 1.17–2.09, *p* < 0.001) and history of drug use (OR: 0.43, 95% CI: 0.28–0.66, *p* < 0.001) were associated with the presence of PTSD, as shown in Table 2.

**Table 2:** Logistic regression model identifying risk factors associated with the presence of PTSD among incarcerated women.

	**Odds Ratio (OR)**	**Lower 95% CI**	**Upper 95% CI**	***P*-value**
Drug use history	0.43	0.28	0.66	<0.001
Violent offense	1.56	1.17	2.09	<0.001

*PTSD, post-traumatic stress disorder*.

### Comparison Between Remission and Non-Remission From PTSD

Among the 220 incarcerated women who suffered from PTSD at imprisonment, 142 spontaneously remitted during their imprisonment, giving a remission rate of 64.5%. The median length of stay in prison was 75.8 (4.0–204.0) months for the remitted individuals and was 38.4 (5.0–168.0) months for non-remitted individuals. As presented in Table 3, incarcerated women with PTSD tended to be remitted if they were elder (41.6 ± 9.2 vs. 37.4 ± 8.7, *p* < 0.01), with violent offense (54.2 vs. 25.4%, *p* < 0.01), physical disease history (56.3 vs. 41.0%), comorbidity with depression (21.1 vs. 1.3%, *p* < 0.01), and a higher proportion in length of sentence longer than 120 months (71.8 vs. 41.0%, *p* < 0.01) compared to those not remitted.

**Table 3:** Comparison between naturally remitted PTSD and non-remitted PTSD among incarcerated women.

**Variables**	**Remitted** **(*****N*** **= 142)**		**Non-remitted** **(*****N*** **= 78)**		***F***	***P*-value**
	***N***	**%**	***N***	**%**		
Residence					1.68	0.20
Urban	82	57.7	52	66.7		
Rural	60	42.3	26	33.3		
Education level					3.00	0.08
Lower	99	69.7	63	80.8		
Upper	43	30.3	15	19.2		
Employment					2.84	0.09
Unemployed	44	31.0	33	42.3		
Employed	98	69.0	45	57.7		
Marriage					0.36	0.55
Married	86	60.6	44	56.4		
Unmarried	56	39.4	34	43.6		
Had kids					0.95	0.33
Absent	30	21.1	21	26.9		
Present	112	78.9	57	73.1		
Criminal history					0.21	0.64
Absent	135	95.1	73	93.6		
Present	7	4.9	5	6.4		
Violent offense					16.69	<0.001
Absent	65	45.8	58	74.4		
Present	77	54.2	20	25.6		
Smoking					3.10	0.08
Absent	121	85.2	59	75.6		
Present	21	14.8	19	24.4		
Drug use history					2.17	0.14
Absent	128	90.1	65	83.3		
Present	14	9.9	13	16.7		
Family history of crime					0.16	0.69
Absent	121	85.2	68	87.2		
Present	21	14.8	10	12.8		
Family history of mental diseases					1.29	0.39
Absent	131	92.3	75	96.2		
Present	11	7.7	3	3.8		
Physical disease					4.72	0.03
Absent	62	43.7	46	59.0		
Present	80	56.3	32	41.0		
Past depression					4.37	0.04
Absent	100	70.4	44	56.4		
Present	42	29.6	34	43.6		
Current depression					16.38	<0.001
Absent	112	78.9	77	98.7		
Present	30	21.1	1	1.3		
Sentence duration (months)					32.73	<0.001
13–60	14	9.9	33	42.3		
61–120	26	18.3	13	16.7		
>120	102	71.8	32	41.0		

*PTSD, post-traumatic stress disorder*.

### Correlates of Remission From PTSD During Imprisonment

The results of the factors associated with remission from PTSD were presented in Table 4. Correlates of remission from PTSD was found in upper education level (OR: 2.64, 95% CI: 1.17–5.96, *p* = 0.02), violent offense (OR: 2.50, 95% CI: 1.12–5.60, *p* = 0.03), history of depression (OR: 0.24, 95% CI: 0.11–0.53, *p* < 0.001), comorbidity with depression (OR: 29.69, 95% CI: 3.50–251.78, *p* = 0.002), and length of sentence (61–120 vs. 13–60 months: OR: 4.2, 95% CI: 1.50–11.75, *p* = 0.006).

**Table 4:** Multivariable regression identifying factors associated with the natural remission from PTSD among incarcerated women.

	**Odds Ratio (OR)**	**95% CI**	***P*-value**
Education level (upper vs. lower)	2.64	1.17–5.96	0.02
Violent offense	2.50	1.12–5.60	0.03
Past depression	0.24	0.11–0.53	<0.001
Current depression	29.69	3.50–251.78	0.002
Sentence duration (61–120 vs. 13–60 mo)	4.2	1.50–11.75	0.006
Sentence duration (>120 vs. 13–60 mo)	1.52	0.54	0.429

*PTSD: post-traumatic stress disorder. Age and length of stay in prison was adjusted in the model*.

## Discussion

### Main Findings

This study examined associated factors of the presence of PTSD at imprisonment and the correlates of spontaneous remission from PTSD in incarcerated women. It found violent offense and no history of drug use were associated with increased risk of the presence of PTSD. Even though most of the incarcerated women with PTSD at imprisonment showed natural remission over a mean length of stay of 76 months in prison, above one-third continued to meet the criteria for the diagnosis. Education level, violent offense, history of depression, comorbid depression, and length of sentence were associated with remission from PTSD. Our study highlights the finding that identification and treatment of PTSD among incarcerated women is underscored. The associated factors with remission from PTSD may facilitate preventing and treating PTSD in prison.

### Relation to Previous Literature

The study found that around two-thirds of the women recovered from their PTSD without specific interventions during years of imprisonment. Previous evidence supported a natural remission from PTSD in most individuals in the community (23, 29). One meta-analysis on the spontaneous remission from PTSD in the community reported remission in 44% over the mean observational period of 40 months (23). In a large study with 8,841 individuals that reported a lifetime remission rate of 92%, more than one half continued to have some symptoms 14 years after onset (29). The variability in the rate of remission from PTSD cannot be explained by the assessed factors as different contexts and time-window were evaluated. Importantly, more than one-third of the women suffering from PTSD persistent over a mean length of stay of 76 months in prison in the current study. This suggests that some individuals are not likely to heal in the absence of treatment and highlights the importance of providing mental health services in prison.

Particular attention should be given to women with PTSD and comorbid depression. Despite not having a clear relationship between current depressive symptomatology and PTSD at admission, comorbid depression seems related to PTSD to form a profile that is much more likely to remit. Previous studies in the community, however, indicated that comorbid affective disorders were linked with a more chronic course of PTSD (30). It is possible that depression and PTSD shared general underlying vulnerability risk factors following the time of trauma (30). In addition, our study showed correlations between past depression and remission from PTSD. Taken together, the link between PTSD and depression in prison is complex. Further prospective research is necessary to assess the course of these disorders over time and the underlying relationship. Albeit the mechanism, interventions should be targeted for those with PTSD and the comorbid symptoms of depression (31).

Our finding that incarcerated women who had violently offended are at higher risk of PTSD consistent with previous studies, which indicated a connection between the perpetration of violence and the development of PTSD in some individuals (32, 33). For example, one meta-analysis showed that about 43% of incarcerated killers and 33% of offenders with other violent crimes developed PTSD after committing offenses (33). This may be caused by the experiences of witness and confronting an event involving serious injury and death when committing the violent offense (32). Additionally, the overlap between violent offenders and individuals developing PTSD may partly arise from the presence of shared risk factors for the two outcomes, such as exposure to traumas prior to prison (34). Previous literature has identified mixed relationships between exposure to traumas, perpetrating violence, and the onset of mental illness (32). This study also found those women were more likely to have their PTSD remit demonstrating the complex role of the violent offense in the course of PTSD. Another finding is that those with longer sentences (i.e., 61–120 vs. 13–60 mo) were more likely to remit while the length of stay was controlled. This is consistent with research suggesting adjustment to prison may lead to improvements in psychological symptoms, such as sleep, appetite, and mood (35). However, some previous studies indicated that PTSD-related symptoms did not decline over time (23), and thus treatment targeting of symptoms is required in an early stage of incarceration. Nevertheless, analyses revealed the length of a sentence longer than 120 months was not associated with remission from PTSD when compared with 13–60 months. Since women with a longer sentence were more likely to be incarcerated with a more severe offense, it is also possible that length of sentence is interacted with violence offense, as it may reflect the seriousness of the offense (36). Therefore, more work should be assigned to explore the role of violent offense and length of sentence in the course of PTSD.

### Implications

This study calls for the routine screening for PTSD among incarcerated women at admission, especially for violent offenders. These results also indicate the importance of treating PTSD in women prisoners. This is evidently true in light of previous findings, the serious adverse outcomes of untreated PTSD (11) and unmet need for mental health care in prison (22). However, there is only modest evidence supporting pharmacological and psychosocial interventions for PTSD in prison contexts (37), and thus more research is required to establish effective treatments.

### Strengths and Limitations

To our knowledge, this is the first retrospective case-control study to examine the natural course of PTSD over the imprisoned period in women by applying systemic and validated instruments. Furthermore, the correlates of existence and remission of the mental health condition in the incarcerated women have also been examined.

There are some limitations to this study. First, the retrospective nature of the self-reporting might allow some inaccurate reporting. The findings should be considered provisional because of the likelihood of recall bias. As a result, the consistency of the findings should be evaluated in future prospective cohort studies. It should be noted that individuals may develop PTSD prior to prison and were diagnosed with PTSD at incarceration. We recommended further research on clear discrimination between individuals who developed PTSD prior to prison and those after incarceration. In addition, we only explored the associations between violent offense and the presence of PTSD. Future studies are necessary to determine the influence of various types of crime on the onset of PTSD. Moreover, as the study focus is on incarcerated women, the generality of these results to other settings (e.g., incarcerated men) remains unknown. However, the study might reflect gender-specific needs and targets for mental health service in prison.

## Conclusions

Follow up of those incarcerated women with PTSD at admission demonstrated that around one-third do not remit from the disorder during a median imprisonment length of 40 months. Comorbid depression, violent offending, and longer sentences appeared to contribute a greater likelihood of remission from PTSD. For the significant numbers whose PTSD does not remit, prompt identification and effective interventions are essential.

## Data Availability Statement

The raw data supporting the conclusions of this article will be made available by the authors, without undue reservation.

## Ethics Statement

The studies involving human participants were reviewed and approved by the Ethical Committees of the Second Xiangya Hospital. The patients/participants provided their written informed consent to participate in this study.

## Author Contributions

SZ, JZ, and XW conceived the study. SZ undertood statistical analysis and drafted the manuscript. XZ collected and cleaned the data. JZ, GM, and XW critically revised the manuscript. All authors contributed to the article and approved the submitted version.

## Funding

The study was supported by the Science and Technology Program of Hunan Province (grant no. 2018SK2133) and Hunan Innovative Province Construction Project (grant no. 2019SK2334).

## Conflict of Interest

The authors declare that the research was conducted in the absence of any commercial or financial relationships that could be construed as a potential conflict of interest.

## Publisher's Note

All claims expressed in this article are solely those of the authors and do not necessarily represent those of their affiliated organizations, or those of the publisher, the editors and the reviewers. Any product that may be evaluated in this article, or claim that may be made by its manufacturer, is not guaranteed or endorsed by the publisher.
